# Dense core vesicle markers in CSF and cortical tissues of patients with Alzheimer’s disease

**DOI:** 10.1186/s40035-021-00263-0

**Published:** 2021-09-26

**Authors:** Neus Barranco, Virginia Plá, Daniel Alcolea, Irene Sánchez-Domínguez, Reiner Fischer-Colbrie, Isidro Ferrer, Alberto Lleó, Fernando Aguado

**Affiliations:** 1 Department of Cell Biology, Physiology and Immunology, Faculty of Biology, University of Barcelona, 08028 Barcelona, Spain; 2 Institute of Neurosciences, University of Barcelona, 08028 Barcelona, Spain; 3 Memory Unit, Department of Neurology, Sant Pau Biomedical Research Institute. Sant Pau Hospital, Autonomous University of Barcelona, 08041 Barcelona, Spain; 4 Center for Networked Biomedical Research on Neurodegenerative Diseases (CIBERNED), 28031 Madrid, Spain; 5 Department of Pharmacology, Innsbruck Medical University, 6020 Innsbruck, Austria; 6 Department of Pathology and Experimental Therapeutics, University of Barcelona, and Bellvitge University Hospital, Bellvitge Biomedical Research Institute, Hospitalet de Llobregat, Spain; 7 Present Address: Center for Translational Neuromedicine, University of Rochester Medical Center, Rochester, NY 14642 USA

**Keywords:** Alzheimer’s disease, Biomarkers, Cerebral cortex, Cerebrospinal fluid, Granulovacuolar degeneration, PCSK1, PCSK2, Tau protein

## Abstract

**Background:**

New fluid biomarkers for Alzheimer's disease (AD) that reveal synaptic and neural network dysfunctions are needed for clinical practice and therapeutic trial design. Dense core vesicle (DCV) cargos are promising cerebrospinal fluid (CSF) indicators of synaptic failure in AD patients. However, their value as biomarkers has not yet been determined.

**Methods:**

Immunoassays were performed to analyze the secretory proteins prohormone convertases PC1/3 and PC2, carboxypeptidase E (CPE), secretogranins SgIII and SgII, and Cystatin C in the cerebral cortex (*n* = 45, provided by Bellvitge University Hospital) and CSF samples (*n* = 66, provided by The Sant Pau Initiative on Neurodegeneration cohort) from AD patients (*n* = 56) and age-matched controls (*n* = 55).

**Results:**

In AD tissues, most DCV proteins were aberrantly accumulated in dystrophic neurites and activated astrocytes, whereas PC1/3, PC2 and CPE were also specifically accumulated in hippocampal granulovacuolar degeneration bodies. AD individuals displayed an overall decline of secretory proteins in the CSF. Interestingly, in AD patients, the CSF levels of prohormone convertases strongly correlated inversely with those of neurodegeneration markers and directly with cognitive impairment status.

**Conclusions:**

These results demonstrate marked alterations of neuronal-specific prohormone convertases in CSF and cortical tissues of AD patients. The neuronal DCV cargos are biomarker candidates for synaptic dysfunction and neurodegeneration in AD.

**Supplementary Information:**

The online version contains supplementary material available at 10.1186/s40035-021-00263-0.

## Background

Alzheimer's disease (AD) is the most prevalent neurodegenerative disorder in the elderly [1]. Because currently there is no effective treatment or prevention for AD, identification of AD fluid biomarkers in the neuropathological progression of the illness is crucial to clinical practice and therapeutic trial design [2–4]. The main clinical symptoms of AD are gradual and progressive memory and cognitive impairments, which strongly correlate with the cortical atrophy-related synaptic and neuronal loss in all affected brain regions [5–7]. Pathologically, AD is characterized by deposits of amyloid-β (Aβ) peptides in extracellular senile plaques and accumulation of hyperphosphorylated tau protein in somatic neurofibrillary tangles and plaque-surrounding dystrophic neurites. In addition to the loss of neural circuitry and neuronal cell bodies, cerebral amyloid angiopathy, granulovacuolar degeneration (GVD), and Hirano bodies with glial activation have also been suggested as part of AD pathology [8].

Reliable cerebrospinal fluid (CSF) signatures have been developed for plaque and tangle pathologies and for associated neurodegenerative processes (reduced Aβ_1–42_ and increased phosphorylated tau (P-tau) and total tau (T-tau) levels, respectively). These core CSF biomarkers are currently routinely used in clinical practice for diagnosis of AD in patients at the mild cognitive impairment or dementia stage of the disease [9]. Additionally, several reports have proposed certain microglial and astrocytic proteins, such as TREM2 and YKL-40, as biomarkers of glial activation in AD [10–13]. Importantly, as decreased synapses are a major quantitative correlate of loss of memory and cognition in AD brains [7], much attention is being focused on biomarkers to detect the degree of synaptic dysfunction and degeneration in early stages of AD. Novel CSF biomarker candidates for synaptic pathology include axonal and pre- and postsynaptic proteins, such as SNAP-25, Syntaxin 1B, neurogranin and neurofilament light chain [14–17].

Other potential CSF biomarkers for synaptic alterations in AD consist of released cargos of the so-called dense core vesicles (DCVs). As synaptic vesicles (SVs), DCVs belong to the regulated secretory pathway in neurons and also, presumably, in astrocytes [18, 19]. In response to membrane depolarization, DCVs release a variety of neuropeptides and growth factors (e.g. brain-derived neurotrophic factor [BDNF]) as well as distinctive molecular components, such as chromogranins (Cg), secretogranins (Sg), and processing enzymes [18]. Although DCVs do not accumulate within synaptic compartments as do SVs, the vast majority of fusion events occur at synaptic boutons and axons [20, 21]. Interestingly, a major and ubiquitous constituent of the DCV matrix, CgA, was one of the first biochemical biomarker candidates for AD synaptic degeneration [22, 23]. Recent advances in proteomic technology have identified secreted DCV proteins in CSF screens for AD patients, such as the prototype granins CgA and CgB and the non-classical granin SgVII (usually called VGF, the nerve growth factor inducible protein VGF) [24, 25]. These observations suggest that DCV proteins may be promising biomarkers of synaptic loss in AD. However, changes in DCV cargos in AD CSF observed in different proteomic and immunological analyses offer variable results, or have not been completely validated [26, 27]. Here, we investigated changes in DCV proteins in the CSF and brain samples of AD patients. Specifically, we examined the neuronal prohormone convertases PC1/3 and PC2, and the neuronal and astroglial carboxypeptidase E (CPE), SgIII and SgII [28–33]. Additionally, we examined the secretory protein Cystatin C (CysC), a neuronal and astrocytic protease inhibitor involved in AD, located mainly in lysosomes and possibly in DCVs [34, 35].

## Materials and methods

### Brain tissues

Post-mortem human AD (*n* = 23) and non-AD (*n* = 22) brain samples (aged 49–86) were obtained from the Institute of Neuropathology Brain Bank IDIBELL Hospital Universitari de Bellvitge (Hospitalet de Llobregat, Spain) under an agreement with the local ethics committee; demographic data are presented in Table 1. Individuals were selected based on the post-mortem diagnosis of AD according to the ABC score (A, Amyloid phases Thal; B, Braak stages of neurofibrillary tangle pathology; C, CERAD stages) following the National Institute on Aging-Alzheimer's Association (NIA-AA) clinical research criteria [36]. The AD-diagnosed subjects corresponded to A3-A4, V-VI (B3) and C3, whereas the non-AD subjects were A0, 0-I/II (B0-BI) and C0.

**Table 1:** Demographic information for Alzheimer's disease and control brains

	Age (years)	Gender	Post-mortem time (hours)	Cortical region	Type of investigation
Control (*n* = 3)	71.0 (± 7.0)	1M/2F	5.2 (± 2.8)	Parietal cortex	Immuno-histochemistry
AD (*n* = 5)	78.4 (± 4.6)	5F	5.7 (± 3.8)		
Control (*n* = 5)	69.6 (± 5.3)	3M/2F	9.3 (± 4.0)	Hippocampus	Immuno-histochemistry
AD (*n* = 4)	75.8 (± 6.5)	2M/2F	5.9 (± 2.2)		
Control (*n* = 7)	73.7 (± 7.3)	4M/3F	6.0 (± 2.8)	Parietal cortex	Western blot
AD (*n *= 7)	80.1 (± 6.6)	2M/5F	6.3 (± 3.4)		
Control (*n* = 7)	73.3 (± 13.1)	7M	6.6 (± 4.7)	Hippocampus	Western blot
AD (*n* = 7)	81.9 (± 3.7)	4M/3F	6.4 (± 1.9)		

*F*, female;* M*, male

### Lumbar CSF samples

Both AD patients and control subjects (healthy volunteers) were recruited from the SPIN cohort in the Memory Unit at the Hospital de la Santa Creu i Sant Pau [37]. This study was approved by the local ethics committee and was carried out in accordance with the Declaration of Helsinki. All patients (or their nearest relatives) and controls gave informed consent to participate in the study. Extensive clinical, neuropsychological, MRI and molecular examinations were performed in all subjects. CSF samples (*n* = 66) were collected by lumbar puncture between 9 am and noon. Centrifugation at 4 °C for 10 min at 2000 × g and storage of 500 μl aliquots in polypropylene tubes at − 80 °C were accomplished within 1 h after collection. All AD patients fulfilled clinical criteria for probable AD according to the revised NIA-AA criteria [38] and had a CSF biomarker profile consisting of decreased Aβ_1-42_ plus high T-tau and P-tau levels (ELISA tests from Innogenetics, Ghent, Belgium), indicating high likelihood of being due to AD. The cut-off values we used to define our AD cohort in this study were 550 pg/ml for Aβ_1-42_, 350 pg/ml for T-tau, and 61 pg/ml for P-tau [39]. The control group was defined according to the following criteria: objective cognitive performance within the normal range (performance within 1.5 standard deviation) on all tests from a specific test battery, clinical dementia rating scale score of 0, no significant psychiatric symptoms or previous neurological disease, and a non-pathological CSF biomarker profile. The average Mini-Mental State Examination (MMSE) score was 21.6 ± 4.4 for AD patients, whereas control subjects had a score of 28 or higher. AD and control groups were well matched for age at CSF collection. Demographic information is presented in Table 2.

**Table 2:** Demographics and CSF profiles of individuals

	Age (years)	Gender	MMSE	APOE allelic frequency	CSF Aβ42 (pg/ml)	CSF tau (pg/ml)	CSF p-tau (pg/ml)	CSF glucose (mmol/l)
Control (*n* = 33)	63.75 (± 7.18)	13M/20F	29.21 (± 0.96)	ε2 = 0.05 ε3 = 0.83 ε4 = 0.12	836.09 (± 154.72)	221.97 (± 57.44)	43.38 (± 10.39)	3.46 (± 0.51)
AD (*n* = 33)	66.94 (± 6.13)	12M/21F	21.58 (± 4.39) ***	ε2 = 0.03 ε3 = 0.44 ε4 = 0.53	353.27 (± 101.20)***	905.32 (± 457.94)***	99.33 (± 31.32)***	3.39 (± 0.51)

Data are presented as mean ± SEM

***P < 0.001, Mann-Whitney test.* F*, female;* M*, male

### Antibodies

Home-made and commercial antibodies against DCV proteins used in this study have been extensively validated for western blot and immunohistochemical methods in previous papers and recognize processed and precursor molecular forms. Polyclonal antibodies against PC2 (LS18 kindly provided by Dr I. Lindberg, University of Maryland) and SgII/Secretoneurin have been described elsewhere [40, 41]. Polyclonal antibodies against PC1/3 were purchased from Abcam (ab3532**,** Abcam Plc, Cambridge, UK) and Thermo (PA1-057**,** Thermo Fisher Scientific, Waltham, MA) and polyclonal antibodies against PC2 were from GeneTex (GTX23533**,** GeneTex Inc, Irvine, CA) [42–44]. Polyclonal antibodies against SgIII (HPA006880, Prestige Antibodies® supported by The Human Protein Atlas) [29, 45] and monoclonal anti-β-actin peroxidase antibodies (AC-15) were purchased from Sigma (Sigma-Aldrich, Diesenhofen, Germany). Monoclonal and polyclonal antibodies against CPE were obtained from BD Biosciences (610758, BD Transduction Laboratories, San Jose, CA) and GeneTex (GTX23533, GeneTex Inc), respectively [45, 46]. Polyclonal antibodies against CysC (ABC20) [47] and monoclonal antibodies against GFAP (MAB3402) were obtained from Millipore Iberica (Madrid, Spain). Monoclonal and polyclonal antibodies against CHMP2B (MAB7509) and CK1δ (AF4568) were purchased from R&D Systems (Minneapolis, MN). Monoclonal antibodies against LAMP-1 (H4A3) and neurofilament light chain (2835) were obtained from Developmental Studies Hybridoma Bank at the University of Iowa (Iowa City, Iowa) and Cell Signaling (Leiden, The Netherlands), respectively. Antibodies against Aβ (M087201-2) and hyperphosphorylated tau (AT8) were from DAKO (Glostrup, Denmark) and Innogenetics (Gent, Belgium), respectively. A complete antibody list is provided in Additional file 1: Table S1.

### Immunohistochemistry

Human brain samples were fixed in 4% paraformaldehyde in 0.1 M phosphate buffer, pH 7.4, by immersion. After being cryoprotected in a 30% sucrose solution, the samples were frozen and sectioned with a cryostat. For peroxidase immunohistochemistry, histological sections were blocked for 30 min in phosphate buffered saline (PBS) supplemented with 10% methanol and 3% H_2_O_2_ and then washed in PBS. Pretreatment with formic acid was used to enhance labeling of plaques. To avoid nonspecific binding, brain sections were blocked in PBS containing 10% serum, 0.2% glycine, 0.1% Triton X-100, and 0.2% gelatin for 1 h at room temperature. Incubations with the primary antibodies were carried out overnight at 4 °C in PBS containing 1% fetal bovine serum, 0.1% Triton X-100 and 0.2% gelatin. Bound antibodies were detected using the avidin–biotin-peroxidase system (Vectastain ABC kit, Vector Laboratories Inc., Burlingame, CA). PBS containing 0.05% diaminobenzidine (DAB) and 0.01% H_2_O_2_ was used to visualize the peroxidase complex. Sections were mounted, dehydrated, and coverslipped in Eukitt® (Sigma-Aldrich, Diesenhofen, Germany). Semiquantitative analysis of CysC immunoreactivity on DAB-stained slices was carried out on the outer and inner layers of the AD and control parietal cortices (*n* = 3, each). Images at 20 × magnification (2 per section, 2 sections per sample) were randomly taken using identical acquisition microscope parameters. The signal intensity of 0.1 × 0.1 mm^2^ fields was measured using the ImageJ® software (NIH, Bethesda, MD). Levels of CysC were initially quantified as arbitrary density units and subsequently AD values were expressed as a percent change from control measurement. Double-labeling fluorescent immunohistochemistry was carried out by incubation with different fluorochrome-conjugated secondary antibodies (Alexa Fluor 488 and Alexa Fluor 568; Molecular Probes, Eugene, OR), and cell nuclei were stained with Hoechst (Sigma-Aldrich, Diesenhofen, Germany). Endogenous autofluorescence was avoided using Sudan Black B (Sigma-Aldrich, Diesenhofen, Germany). Sections were mounted in Mowiol (Merk Chemicals Ltd., Nottingham, UK) and visualized with a Leica TCS SPE scanning confocal microscope. Colocalization analyses of DCV proteins with GVD markers were performed by ImageJ software, using the threshold-corrected Mander’s correlation coeficient, which ranges between 1 (high-colocalization) and 0 (low-colocalization). The specificity of the immunolabeling was checked omitting primary antibodies, using nonspecific IgG instead of them and using a previous incubation of the primary antibodies with an excess of antigen (Proteintech Group Inc., Deansgate, Manchester, UK). No immunostaining was observed in these conditions.

### Western blotting and radioimmunoassay

Equal volumes of lumbar CSF samples were collected in vials and brain tissues were homogenized in ice-cold lysis buffer containing 50 mM Tris–HCl, pH 7.4, 150 mM NaCl, 5 mM MgCl_2_,1 mM EGTA, 1% Triton X-100, and a protease inhibitor cocktail (Roche Diagnostics GmbH, Mannheim, Germany). The CSF samples and postnuclear brain lysates were mixed with 3 × sample buffer (188 mM Tris–HCl, 30% glycerol, 3% SDS, 0.01% Bromophenol Blue, and 15% β-mercaptoethanol), electrophoresed in 10%–12% SDS-acrylamide gel (Bio-Rad Laboratories, Hercules, CA), and then transferred to polyvinylidene difluoride immobilization membranes (Whatman® Schleicher & Schuell, Keene, NH). Tissue results were normalized for total protein content (data obtained from Ponceau staining scans). Membranes were blocked in 5% nonfat milk powder solution in Tris-buffered saline and Tween-20 (TBST; 140 mM NaCl, 10 mM Tris/HCl, pH 7.4 and 0.1% Tween-20) for 1 h at room temperature and then incubated with primary antibodies in blocking solution overnight at 4 °C. After numerous washes in blocking buffer, the membranes were incubated for 1 h with horseradish peroxidase-conjugated secondary antibodies (Bio-Rad Laboratories, Hercules, CA). Enhanced chemiluminescence reagents ECL™ (GE Healthcare, Buckinghamshire, UK) and X-ray films (AGFA) were used to visualize bound antibodies. Blot images were taken with a scanner and densitometric values were obtained using a Java-based image processing software (ImageJ® software). The amount of total proteins in brain homogenates (10–15 µg) and CSF volume (2–5 µl) and exposure times of the films were empirically determined to obtain the adequate linear range for quantitative analysis. For protein forms with different electrophoretic mobilities, a single average exposure of the film is shown in the figures, whereas quantitative analysis of each form was performed at different and suitable amounts of total protein/volume and exposure time of the films.

The SgII/secretoneurin radioimmunoassay was performed as described previously [41]. In brief, samples were denatured to avoid protein degradation and antiserum was added in the radioimmunoassay buffer and incubated for 24 h at 4 ºC. Then iodinated secretoneurin (200 cpm/μL) was added and samples were incubated for an additional 24 h at 4 ºC. The non-bound tracer was separated by dextran-coated charcoal absorption and pulled down by centrifugation. The iodinated tracer remaining in the supernatant fraction was quantified, with a detection limit of 1–2 fmol.

### Statistical analysis

Quantitative data were statistically analyzed using GraphPad Prism 6.01® software (GraphPad Software, San Diego, CA) with *t*-test and Mann–Whitney test. All data are presented as the means ± SEM. Significance was set at *P* < 0.05. Correlations of the measured values were examined using the Bonferroni-corrected Spearman correlation coefficient. A significance cutoff of *P* ≤ 0.0019 based on Bonferroni correction of multiple comparisons and *P* ≤ 0.05 in non-corrected comparisons were applied.

## Results

### DCV markers are neuropathologically accumulated in dystrophic neurites and GVD bodies in the AD cerebral cortex

To examine DCV changes in the cerebral cortex of AD patients, we performed Western blotting and immunostaining analyses in homogenates and histological sections from AD patients and age-matched controls. PC1/3, PC2, CPE, SgIII and SgII were abundantly found by immunoblotting in cortical samples (Additional file 1: Fig. S1). In addition to the unprocessed and mature forms, higher and lower electrophoretic mobility bands were detected with polyclonal antibodies against DCV proteins, likely corresponding to the described aggregated and cleaved species [30–32]. A robust detection for all of these proteins was noted in CSF samples, essentially their precursor and mature forms. Volumes as small as 1 µL were enough to detect some of these proteins in the CSF (i.e. SgIII). As previously reported, only the monomeric form of the secretory protease inhibitor CysC was detected in the CSF (~ 14 kDa), whereas cortical tissues also displayed oligomeric/aggregated species [48, 49]. When performing analyses of similar CSF volumes, levels of membrane and cytosolic proteins such as synaptophysin (Syp) and β-actin (Additional file 1: Fig. S1), as well as the potential AD biomarker neurofilament light chain (data not shown), were under the detection limit. Thus, besides the abundance in brain tissues, DCV proteins are broadly detected in the CSF, feasibly corresponding with released species.

**Fig. 1:**
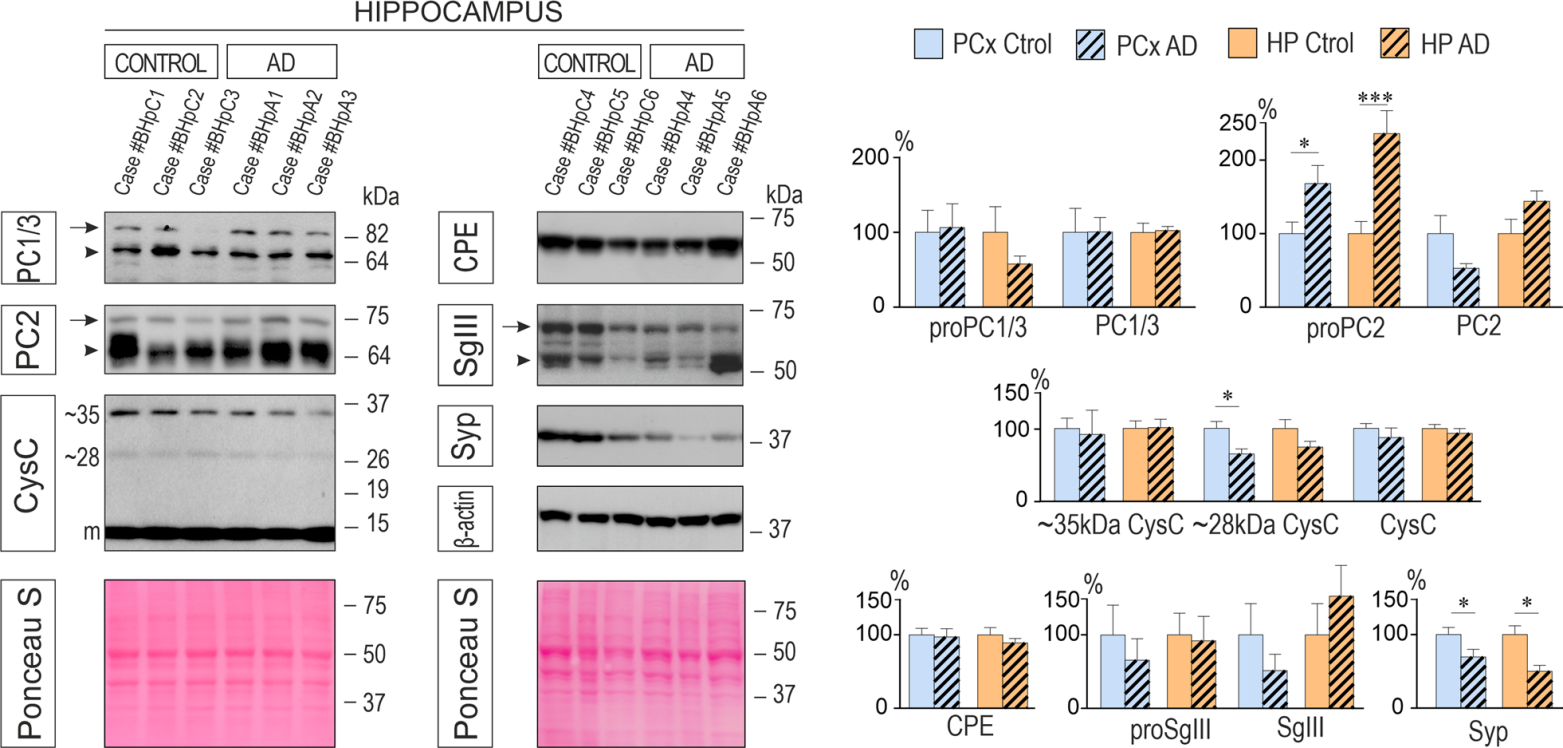
Levels of secretory proteins in the AD cerebral cortex. *Left* Representative western blots showing protein levels of PC1/3, PC2, CPE, SgIII, CysC, Syp, and β-actin in homogenates of control and AD hippocampus. Each of the two immunoblotting columns illustrates levels of different proteins in the same cases blotted on the same membrane. The total membrane-transferred proteins were detected by Ponceau S staining. The mobility of molecular mass markers (in kDa) is indicated. *Right* Secretory (PC1/3, PC2, CPE, SgIII, CysC) and synaptic vesicle (Syp) protein levels in the parietal cortex (PCx) and hippocampus (HP) of AD patients compared to the age-matched controls (*n* = 7 for each tissue). Graphs show the percentage variation compared with controls and normalized to total membrane-blotted proteins. Levels of the precursor form of PC2 (proPC2) were increased in AD cortical tissues, whereas CysC dimers/aggregates were reduced in the PCx of AD patients. A consistent decrease of Syp is detected in both AD cortices, HP and PCx. Data are presented as mean ± SEM. *P < 0.05; **P < 0.01; ***P < 0.001, Mann–Whitney test

Next, we examined the levels of distinct molecular forms of secretory proteins in AD brains by immunoblotting (*n* = 7 per tissue and condition) (Fig. 1 and Additional file 1: Fig. S2). Comparing DCV protein amounts (normalized to membrane-transferred total protein) in hippocampus and parietal cortex of AD cases with controls, we only found changes for the 72-kDa precursor form of PC2 (proPC2). The proPC2 species was increased 1.7-fold in parietal cortex (*P* = 0.038) and 2.4-fold in hippocampus (*P* = 0.0006). Interestingly, the ~ 28 kDa form of CysC was significantly decreased in the parietal cortex of AD patients (35%, *P* = 0.038). This electrophoretic band probably corresponds to dimeric or aggregated forms of CysC, which have been associated with amyloid damage [48, 49]. Finally, a prominent reduction was evident in the content of the SV-specific integral protein Syp both in parietal cortex (30%, *P* = 0.042) and in hippocampus (50%, *P* = 0.017) of AD patients. These results show that the global levels of different DCV markers are mostly preserved in AD cortices, whereas ubiquitous SV proteins are dramatically reduced.

**Fig. 2:**
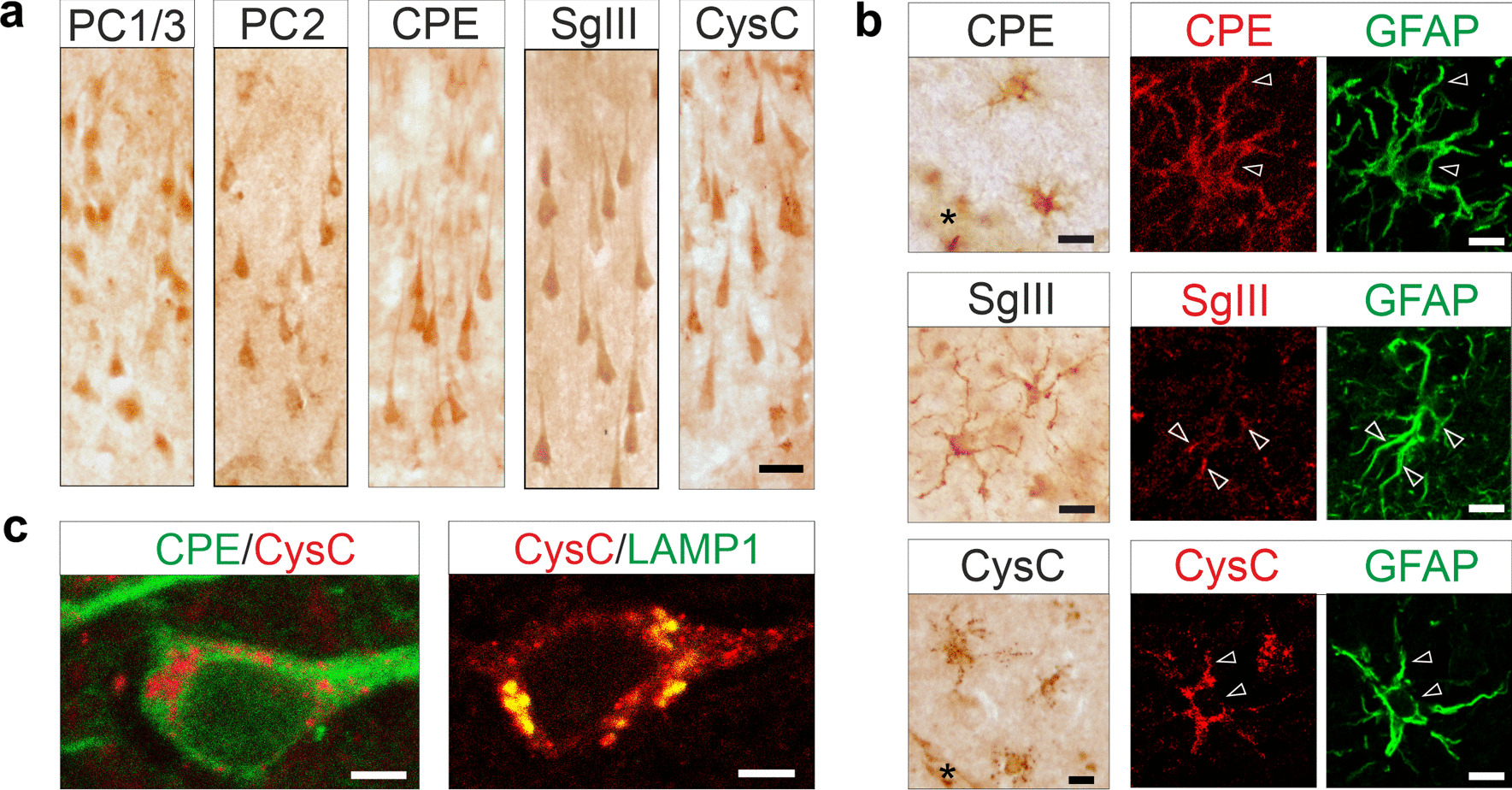
Distribution of secretory proteins in the control cerebral cortex. **a** Peroxidase immunohistochemistry shows distribution of representative secretory proteins (PC1/3, PC2, CPE, SgIII and CysC) in the CA1 region of the hippocampus. Scale bar, 20 μm. **b** Peroxidase immunohistochemistry and double confocal immunofluorescences illustrating SgIII (grey matter), CPE and CysC (white matter) in neocortical astrocytes. Arrowheads indicate glial soma and projections. Scale bars, 10 μm. **c** Double immunofluorescences of two different neocortical pyramidal neurons showing CysC colocalization with the lysosomal marker LAMP1, but not with the DCV cargo CPE. Scale bars, 5 μm

**Fig. 3:**
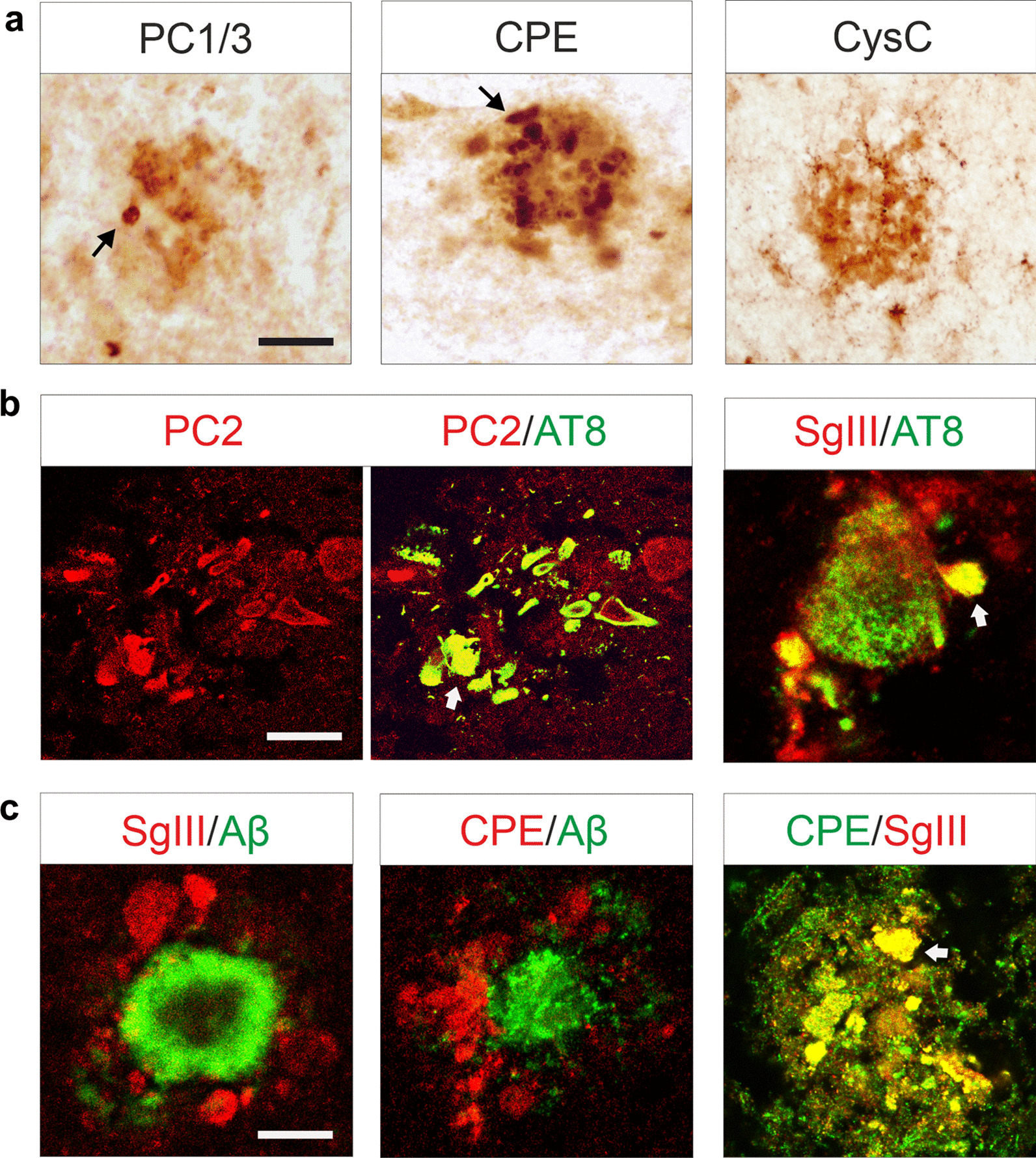
Distribution of secretory proteins in the AD cerebral cortex. **a** Representative peroxidase immunohistochemistry for different secretory proteins in senile plaques at the superficial layers of the AD parietal cortex. Dystrophic neurite-like structures are indicated by arrows. Scale bar 20 µm. **b** In the same cortical senile plaques, AT8 immunofluorescence reveals PC2^+^ and SgIII aberrant structures as dystrophic neurites. Arrows indicate colocalization in yellow. Scale bar, 20 µm. **c** Three different double immunofluorescence images showing focal Aβ^+^ deposit surrounded by positive SgIII and CPE accumulations and the presence of SgIII and CPE in the same aberrant structures. Arrows indicate colocalization in yellow. Scale bar 20 µm

To examine the alterations of DCV proteins in situ, we performed peroxidase immunohistochemical and confocal immunofluorescence analyses in brain sections from control (*n* = 8) and AD cases (*n* = 9). In controls, PC1/3, PC2, CPE, SgIII, SgII and CysC were widely distributed throughout the cerebral cortex, consistent with previous studies [45, 50–52] (Fig. 2). All of these proteins were abundant in neuronal populations of the neocortex and hippocampus (Fig. 2a), although a differential and robust immunolabeling for CPE was detected filling dendritic shafts (Fig. 2a, c). Immunostaining for SgIII, CPE, and CysC was also detected in GFAP^+^ astrocytes (Fig. 2b), whereas prohormone convertases were absent in glial cells. In neurons, subcellular structures labeled for secretory proteins included somatic and dendritic granules, varicose fibers, and terminal-like buttons, as we previously reported [45]. Granular compartments immunostained for typical DCV markers were negative for the lysosomal marker LAMP1 (not shown), whereas CysC immunofluorescence was largely associated with LAMP1^+^ structures (Fig. 2c). Immunostaining alterations for secretory proteins in AD cortices were mainly associated with senile plaques (Fig. 3a). All typical DCV components were aberrantly accumulated, to different degrees, in AT8^+^  dystrophic neurites surrounding amyloid plaques, in the parietal cortex and hippocampus (in each examined AD cases) (representative examples are shown in Fig. 3b, c). In some samples, intense immunolabeling for SgIII and CysC was detected in reactive astrocytes close to the amyloid deposits (data not shown), as described elsewhere [45, 51]. No major changes were detected for secretory proteins in non-plaque areas of the AD brains, including tangle-bearing neuronal somata identified by the AT8 antigen (excepting cells showing GVD, see below). Only a slight, but consistent, increase in CysC was found in pyramidal neurons located in outer and inner layers of the AD parietal cortex (Additional file 1: Fig. S3).

**Fig. 4:**
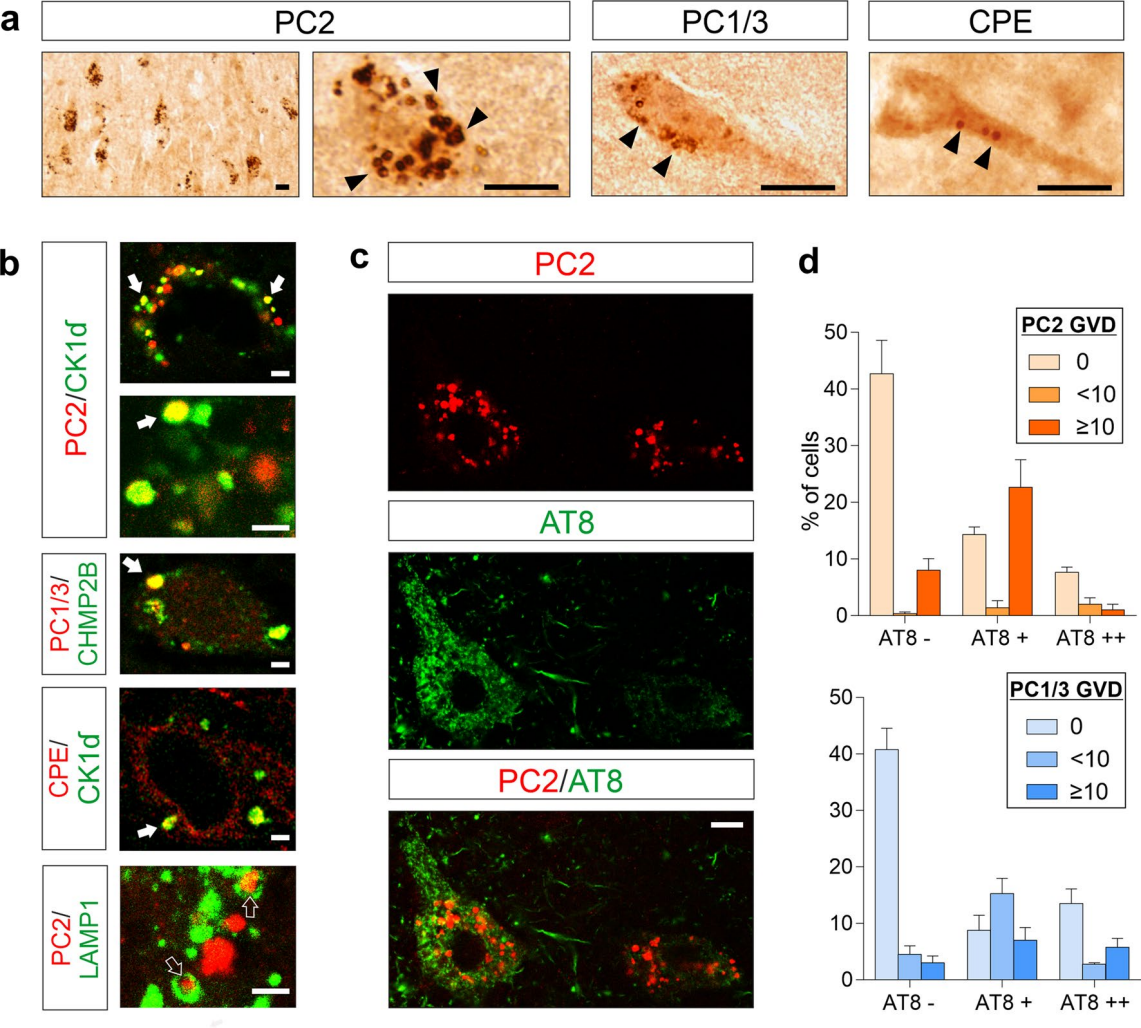
DCV proteins are accumulated in GVD bodies in CA1 pyramidal neurons of AD patients. **a** Peroxidase immunolabelling shows PC2, PC1/3 and CPE accumulations in aberrant granules (arrowheads) located in CA1 pyramidal neurons of AD hippocampus. Scale bars, 10 μm. **b** PC2, PC1/3 and CPE colocalization with the markers of GVD bodies CK1δ and CHMP2B (arrows) and the presence of lumenal PC2 and the transmembrane LAMP1 in the same AD granule (open-arrows) in different neuronal cell bodies of AD cases. Scale bars, 2 μm. **c** PC2^+^ GVD inclusions are found either in mature (AT8^++^) (cell at left) or in undeveloped (AT8^+^) (cell at right) neurofibrillary tangles in CA1 pyramidal neurons of an AD case. Scale bar, 7 μm. **d** Graphs showing the percentages of cells displaying absent (0), few (< 10), or many (≥ 10) PC2- and PC1/3-labelled GVD in AD cases, depending on the occurrence of neurofibrillary tangles (negligible, AT8^−^; low, AT8^+^, strong, AT8^++^)

Unexpectedly, we found GVD-shaped structures immunolabelled for some DCV proteins in CA1 pyramidal somata (and to a lesser extent in CA2 and subiculum) in each of the four examined AD hippocampi (Fig. 4a). These granules had strong immunostaining for PC2 and moderate immunostaining for PC1/3, whereas only one AD case displayed scarce CPE^+^ large granules (Fig. 4a). An average of 241 ± 4 CA1 pyramidal neurons containing aberrant PC2^+^ granules per mm^2^ was observed in AD (*n* = 4), whereas age-matched controls were virtually devoid of these (a few cells, and in only 1 out of 5 cases). Double labeling of PC1/3, PC2 or CPE and the established GVD markers CK1δ and CHMP2B substantiated the neuropathological identity of these large granules (Fig. 4b). Mander’s correlation coefficient for CK1δ overlapping with PC2 was 0.55 ± 0.13 and for PC2 overlapping with CK1δ was 0.62 ± 0.20 (*n* = 12), whereas Mander’s coefficient for CHMP2B overlapping with PC1/3 was 0.43 ± 0.25 and for PC1/3 overlapping with CHMP2B was 0.54 ± 0.28 (*n* = 10). These values indicate good colocalization between DCV proteins and GVD bodies in the AD hippocampus. Additionally, an atypical colocalization of PC2 and PC1/3 with the lysosomal marker LAMP1 was also detected in GVD bodies (Fig. 4b). Interestingly, the PC2- and PC1/3-immunolabelled GVD inclusions were associated with neurofibrillary tangle pathology in AD samples (Fig. 4c, d). Most pyramidal neurons displaying GVD bodies positive for PC2 (81%) and PC1/3 (77%) also contained hyperphosphorylated tau (AT8^+^). Although the GVD inclusions positive for DCV proteins were detected in neurons containing mature neurofibrillary tangles, the DCV-labeled aberrant granules were more often present in early-stages of neurofibrillary tangle formation (Fig. 4c, d).

Taken together, these results show that an aberrant accumulation of DCV components is associated with two neuropathological hallmarks of the AD cerebral cortex, plaque-related dystrophic neurites and GVD bodies.

### Decreased levels of secretory DCV cargos in CSF from AD patients are associated with neurodegeneration markers

**Fig. 5:**
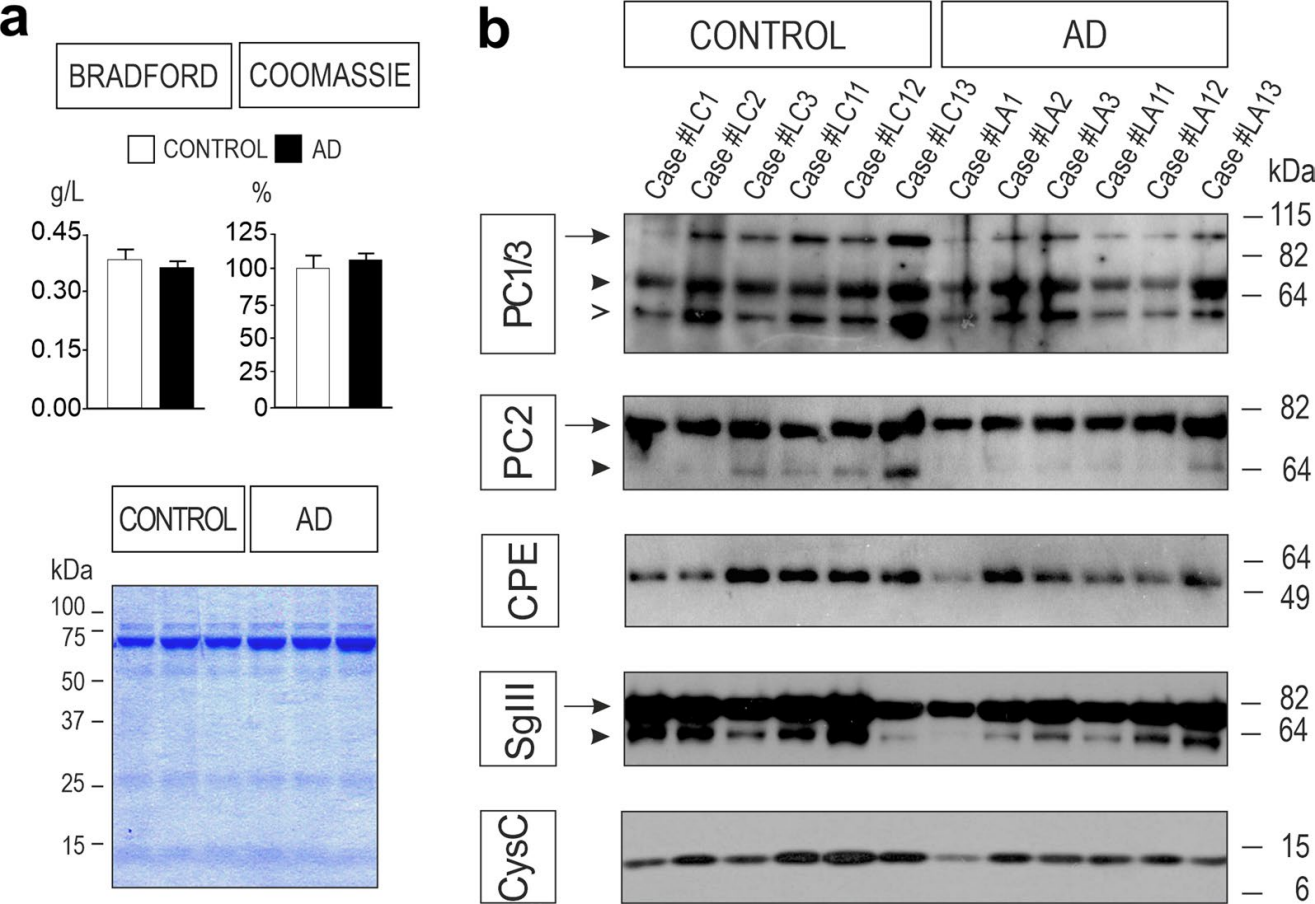
Western blot analysis of secretory proteins in the CSF of AD patients. **a** Total protein content in control and AD CSF samples was determined by Bradford assay and Coomassie staining of SDS-PAGE gels. **b** Representative immunoblots show levels of PC1/3, PC2, CPE, SgIII, and CysC in the same control or AD CSF samples. Arrows and arrowheads indicate unprocessed and mature protein forms, respectively, and the symbol > points to a lower-electrophoretic-mobility form

**Fig. 6:**
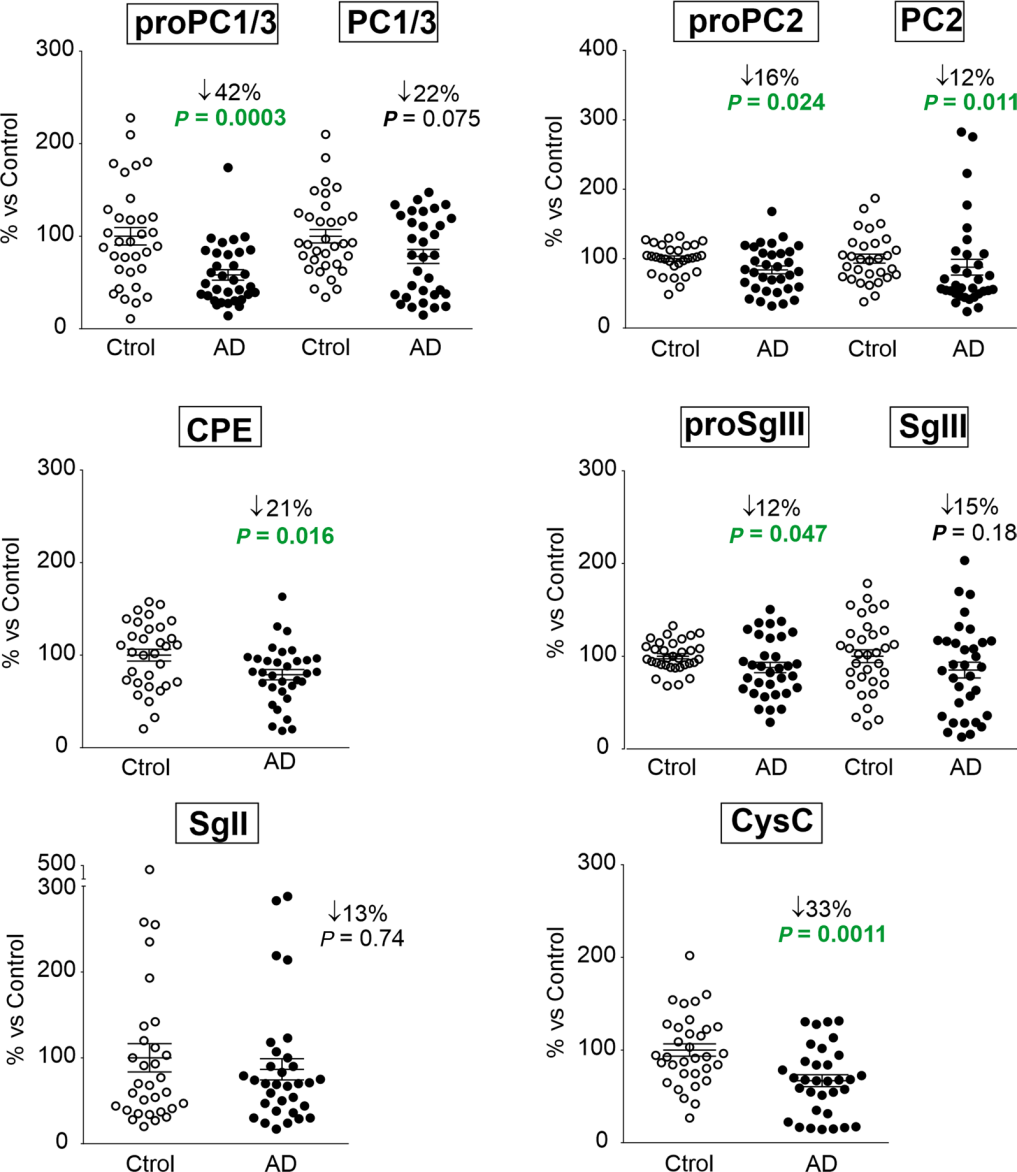
CSF samples from AD patients display decreased levels of secretory proteins. PC1/3, PC2, CPE, SgIII and CysC were analyzed with Western blot and SgII with radioimmunoassay. Scatter dot-plots represent percent variation of secretory protein levels in the CSF of AD patients (*n* = 33) compared to controls (*n* = 33). Data are presented as the mean ± SEM of analysis. Statistically significant difference was calculated using a two-tailed Mann–Whitney test

Next, we investigated the levels of DCV proteins in 66 CSF samples from phenotypically well-characterized AD patients (*n* = 33) and cognitively normal controls (*n* = 33) with immunoblotting and radioimmunoassay methods. No differences in the CSF total protein content were found between AD and controls, either by Bradford assay or Coomassie staining of SDS-PAGE gels (Fig. 5a). However, levels of most molecular forms of DCV proteins were decreased in the CSF from AD patients compared with those from cognitively normal controls (Figs. 5b and 6). Representative immunoblots for different secretory proteins of the same AD and non-AD individuals are illustrated in Fig. 5b. The most prominent reduction in AD was found for unprocessed PC1/3 (− 42% ± 5.7%, *P* = 0.0003). Although levels of the mature form were apparently lower in AD, they did not reach statistical significance (*P* = 0.075) (Fig. 6). When we quantified the levels of mature and unprocessed forms of PC1/3 together, a significant decrease was detected in the AD group (− 24%, *P* = 0.03). Consistently, we also detected an apparent PC1/3 cleaved form displaying a lower electrophoretic mobility identified by the polyclonal antibodies used in this study. When analyzing this molecular species together with the other two bands, we found decreased levels of total PC1/3 in the CSF from AD patients compared to controls (*P* = 0.05). In addition, each of the two bands detected by PC2 antibodies that represent precursor and mature forms, was statistically decreased in AD samples (− 16%, *P* = 0.024 and − 12%, *P* = 0.011, respectively; Fig. 6), as well as in a pooled quantification (− 18%, *P* = 0.0009). The DCV-contained exopeptidase CPE was reduced by ~ 20% in the CSF of the patient cohort (*P* = 0.016). Regarding members of the granin family, the most abundant form of SgIII in the CSF, proSgIII, was decreased in AD (*P* = 0.047), whereas no significant differences were detected for the processed protein. An analysis of the two forms showed no differences between AD and non-AD individuals (*P* = 0.612). We also determined the CSF levels of SgII/secretoneurin with an radioimmunoassay assay. No statistical differences were found between AD and control groups. Finally, a reduction of about 33% was observed for the secretory protein CysC in the CSF of AD patients (*P* = 0.001).

**Fig. 7:**
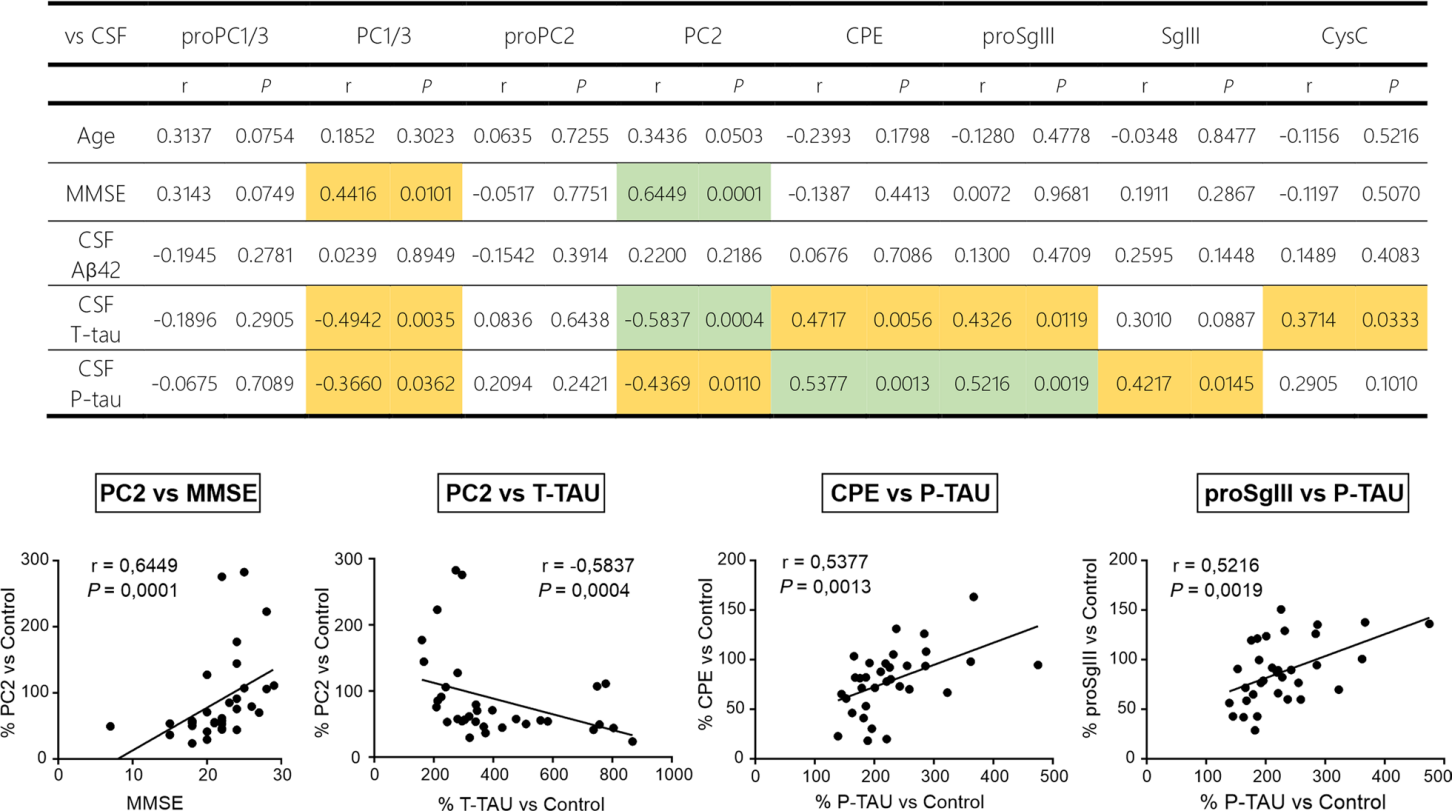
Correlation analysis of CSF secretory proteins with age, cognitive performance, and core CSF biomarkers in AD subjects. *Top* Table summarizing Bonferroni-corrected Spearman correlations of secretory protein forms with age, MMSE score, Aβ1–42, P-tau, and T-tau. Significant correlations (*P* < 0.0019) are highlighted in green. Non-corrected significant correlations (*P* < 0.05) are highlighted in yellow. *Bottom* Significant correlations are represented in scatter plots

To investigate the possible relationships of secretory protein forms with age, cognitive status (MMSE), and the core CSF biomarkers of AD (Aβ_1–42_, P-tau and T-tau), Spearman correlation analysis was carried out in the AD cohort (33 individuals) (Fig. 7). No molecular forms of secretory proteins were significantly correlated with age or Aβ_1–42_ (Fig. 7). In contrast, we detected strong correlations, positive between the mature PC2 form and MMSE score (*P* = 0.0001, *r* = 0.6449) and inverse between PC2 and T-tau (*P* = 0.0004, *r* =  − 0.5837), applying Bonferroni correction. Non-corrected analysis further evidenced an inverse correlation of P-tau with PC2 and PC1/3 and T-tau with PC1/3, and a direct correlation of MMSE with PC1/3. Additionally, corrected analysis evidenced positive correlations between levels of P-tau and CPE (*P* = 0.0013, *r* = 0.5377) and the precursor form of SgIII (*P* = 0.0019, *r* = 0.5216), whereas non-corrected analysis showed a direct correlation of levels of CPE, CysC and different forms of SgIII with P-tau and T-tau (Fig. 7).

## Discussion

The present findings show that the levels of neuronal prohormone convertases and other DCV proteins decrease in the CSF of AD patients, and that this decrease correlates both with the cognitive impairment status and with established CSF neurodegenerative markers. In AD brains, these proteins accumulate in plaque-surrounding dystrophic neurites and reactive astroglia, whereas PC1/3, PC2 and CPE are also remarkably accumulated in hippocampal GVD bodies.

In contrast to the global loss of major SV components (e.g. Syp and Rab3A) [53, 54], we show that the total levels of DCV proteins remain mostly invariable in the cerebral cortex of AD patients. Only proPC2 shows a reliable increase in AD samples, mainly in the hippocampus. These observations support our previous data by Plá et al. [45] on SgIII and CPE, but differ from others [25, 52, 55]. Yakovleva et al. [55] have reported an increase in the PC2 form in AD parietal cortex, whereas no changes of PC1/3 and PC2 or decrease of PC1/3 has been shown in the injured frontal cortex [25, 52]. Because SV markers are virtually restricted to presynaptic terminals, their global alterations likely reflect variations in synaptic contacts. However, the broad distribution of DCV proteins makes it impossible to associate their total levels with synaptic changes. In fact, in situ immunodetection (Figs. 2, 3, 4) reveals dramatic alterations of these markers at cellular and subcellular levels that are hidden in global analyses (Fig. 1 and Additional file 1: Fig. S2).

In the AD cerebral cortex, we found no apparent changes in DCV proteins in normal-shaped axons and terminal-like buttons. However, we detected accumulations of DCV cargos in somata and projections of neurons and astrocytes associated with the degenerative and inflammatory progression of the disease. Remarkably, we detected deposits of DCV components (mainly PC2 and PC1/3) in GVD bodies in pyramidal neurons in AD brain. These inclusions are believed to be aberrant lysosomes, exhibiting a central dense core, located in neurons of hippocampus and other brain areas in patients with different primary tauopathies, especially in AD [56–58]. Our results showing LAMP1-surrounded PC2^+^ inclusions in cells that contain immature neurofibrillary tangles are consistent with the idea that the emergence of GVD granules precedes the appearance of fully-formed tangles in degenerating neurons [56]. To our knowledge, the present study is the first to provide evidence of a DCV cargo in these neuropathological granules. A plethora of proteins has been identified in GVD bodies [56, 59–61], including cytoskeletal components and proteins of the unfolded protein response, autophagic and transduction pathways, cell stress and apoptosis, the necrosome complex, and the RNA-binding proteins, but not DCV components. Therefore, our results involve the regulated secretory pathway in AD-related GVD. This is supported by reports showing the presence of the SV protein TMEM230, the Golgi apparatus Golgin A4, and components of the endoplasmic reticulum unfolded protein response in GVD bodies [56, 61–63]. Because in addition to the hippocampus, the parietal cortex can also display GVD [58], our results of elevated levels of the precursor form of PC2 in AD homogenates point to non-processed PC2 as the main form in GVD bodies, which may be related to the high tendency of proPC2 to form large aggregates [64].

Another neuronal degenerative structure displaying prominent alteration of DCV cargos is the dystrophic neurite. Since the report of striking amounts of CgA in AD dystrophic neurites, almost every DCV marker has been detected as accumulating in these degenerative structures, including transmitter cargos (e.g. BDNF) and intrinsic molecular machinery (i.e. granin family members) but not SV components [22, 45, 50, 54, 65–67]. In this study, we show the previously unrevealed accumulation of PC1/3 and PC2 in amyloid-surrounding dystrophic neurites. Thus, dystrophic neurites are a significant location for DCV retention. The recent work of Sadleir et al. [68] showing a dramatic microtubule and trafficking disruption in dystrophic neurites associated with amyloid plaques strongly substantiates the aberrant accumulation of DCVs in these swollen structures and, therefore, suggests an important impairment in their vesicular cargo release. Lastly, in addition to the neuronal changes, the increased immunostaining of SgIII and CysC in peri-plaque astrocytes is consistent with previous studies and likely corresponds with transcriptional changes in activated cells and/or an Aβ-induced deficiency in glial secretion [29, 33, 45, 51, 69].

In the CSF of AD patients, we detected that five out of the six analyzed proteins are significantly lowered compared with controls. Decreased levels of PC1/3, PC2 and SgIII have very recently been found in large-scale proteomic CSF screens from AD patients, but this has not been immunologically verified yet [24, 70–74], whereas variable results for CysC, CPE and SgII have been obtained [24, 71, 75–78]. In general, decreased levels of different DCV cargos (i.e. CgA, VGF and 7B2) have been found in AD CSF, with only a few reports showing opposing results [26, 78]. Several explanations might be offered for these discrepancies. First, the work of Duits et al. [78], illustrating high levels of CgA, SgII and VGF in CSF of patients with mild cognitive impairment progressing to AD, suggests that early events in the AD pathophysiological cascade can display opposite changes in DCV cargos in later stages. This agrees with earlier studies showing that the synaptic decline in early stages of AD is compensated by an increase in synaptic size of the remaining contacts [5]. Therefore, failure in peptidergic neurotransmission, presumably occurring in dystrophic neurites, could trigger compensatory production of DCVs in unaffected terminals. In addition, taking into account that cargos such as CPE, SgIII and CysC are robustly produced by neurons and non-neuronal cells, CSF levels could depend on the balance between neuronal impairment and glial activation in different AD stages.

Since the CSF likely reflects the composition of the extracellular milieu in the central nervous system, decreased levels of secretory proteins in the CSF of patients could contribute to the pathophysiology of the disease. Based on experimental evidence, low levels of neuroactive peptides, such as VGF and BDNF, may contribute to memory and cognitive impairments, whereas decreased levels of CysC and the prohormone convertase-binding proteins 7B2 and proSAAS could promote amyloidogenesis [34, 66, 67, 79, 80]. Several mechanisms may be put forward to underlie the occurrence of low concentrations of secretory proteins in AD CSF. Although alterations in the blood–brain barrier and in extracellular peptide degradation cannot be ruled out, both transcriptional changes and release impairments may underlie the decline of secretory components in AD CSF [25, 33, 68, 81–83]. Additionally, the decreased levels of DCV proteins in AD CSF may reflect the synaptic and neuronal loss occurring in the disease. The present results of robust inverse correlation of the neuronal prohormone convertases with tauopathy markers and cognitive impairment strongly suggest that the reduced levels of these DCV cargos correspond with the spreading neural damage. Conversely, the levels of proteins expressed by both neurons and astrocytes (e.g. SgIII) directly correlate with neurodegeneration markers in AD. Within the AD cohort, besides impairment of the neuronally produced forms, it is conceivable that production of secretory proteins by activated astrocytes increases as neuronal damage worsens [29, 45, 51, 69]. Furthermore, the connection of secretory proteins with neurodegeneration, but not with amyloid plaque pathology, is consistent with recent proteomic studies in non-AD neurodegenerative diseases, such as frontotemporal dementia, where a decline of CgA, VGF and CysC occurs in the CSF of patients [84–86].

The current diagnosis methods for AD include cognitive tests, neuroimaging techniques and CSF assays. However, there is still no clinical strategy available for the precise and early detection of AD. Some limitations involve disease heterogeneity, co-morbidities and overlapping between different neurodegenerative disorders [87]. Among new potential biomarkers, those that monitor synaptic alterations are needed for prognosis of disease progression and evaluation of effects of drugs on AD [88]. Because the altered DCV proteins in CSF have been implicated in synaptopathies beyond neurodegenerative diseases, such as delirium and schizophrenia [89, 90], DCV cargos could be used as complementary biomarkers to follow synaptic dysfunction and loss and neurodegeneration in AD.

## Conclusions

The current results show alterations of DCV cargos belonging to regulated secretory pathways, in the CSF and cortical tissues of AD patients. Because neuron-specific PC1/3 and PC2 are associated with tauopathy and cognitive impairment, we propose these proteins as complementary biomarker candidates for tracking AD-associated synaptic dysfunction and neurodegeneration.

## Supplementary Information

**Additional file 1: Fig. S1**. Brain secretory proteins are abundantly detected in human CSF. **Fig. S2**. Levels of secretory proteins in the AD parietal cortex. **Fig. S3**. Levels and distribution of CysC in the AD parietal cortex. **Table S1**. List of used primary antibodies.

## Data Availability

All data generated or analyzed during this study are included in this article and its supplementary materials.

## Abbreviations

Aβ: Amyloid-β
AD: Alzheimer's disease
BDNF: Brain-derived neurotrophic factor
Cg: Chromogranin
CHMP2B: Charged Multivesicular Body Protein 2B
CK1δ: Casein kinase 1 delta
CPE: Carboxypeptidase E
CSF: Cerebrospinal fluid
CysC: Cystatin C
DCV: Dense core vesicle
GFAP: Glial fibrillary acidic protein
GVD: Granulovacuolar degeneration
LAMP-1: Lysosomal associated membrane protein 1
MMSE: Mini-mental state examination
PBS: Phosphate buffered saline
PC: Prohormone convertase
TMEM230: Transmembrane protein 230
TREM2: Triggering receptor expressed on myeloid cells 2
P-tau: Phosphorylated tau
T-tau: Total tau
Sg: Secretogranin
SNAP-25: Synaptosomal-associated protein of 25 kDa
Syp: Synaptophysin
SV: Synaptic vesicle
VGF: The nerve growth factor inducible protein VGF
