# Supplementary material for: Dense core vesicle markers in CSF and cortical tissues of patients with Alzheimer’s disease
Source: Transl Neurodegener. 2021 Sep 26;10:37. doi: 10.1186/s40035-021-00263-0 (PMC8466657; doi:10.1186/s40035-021-00263-0)
Supplement: Supplementary file 1 — Additional file 1: Fig. S1. Brain secretory proteins are abundantly detected in human CSF. Fig. S2. Levels of secretory proteins in the AD parietal cortex. Fig. S3. Levels and distribution of CysC in the AD parietal cortex. Table S1. List of used primary antibodies. [file 40035_2021_263_MOESM1_ESM.docx]

**
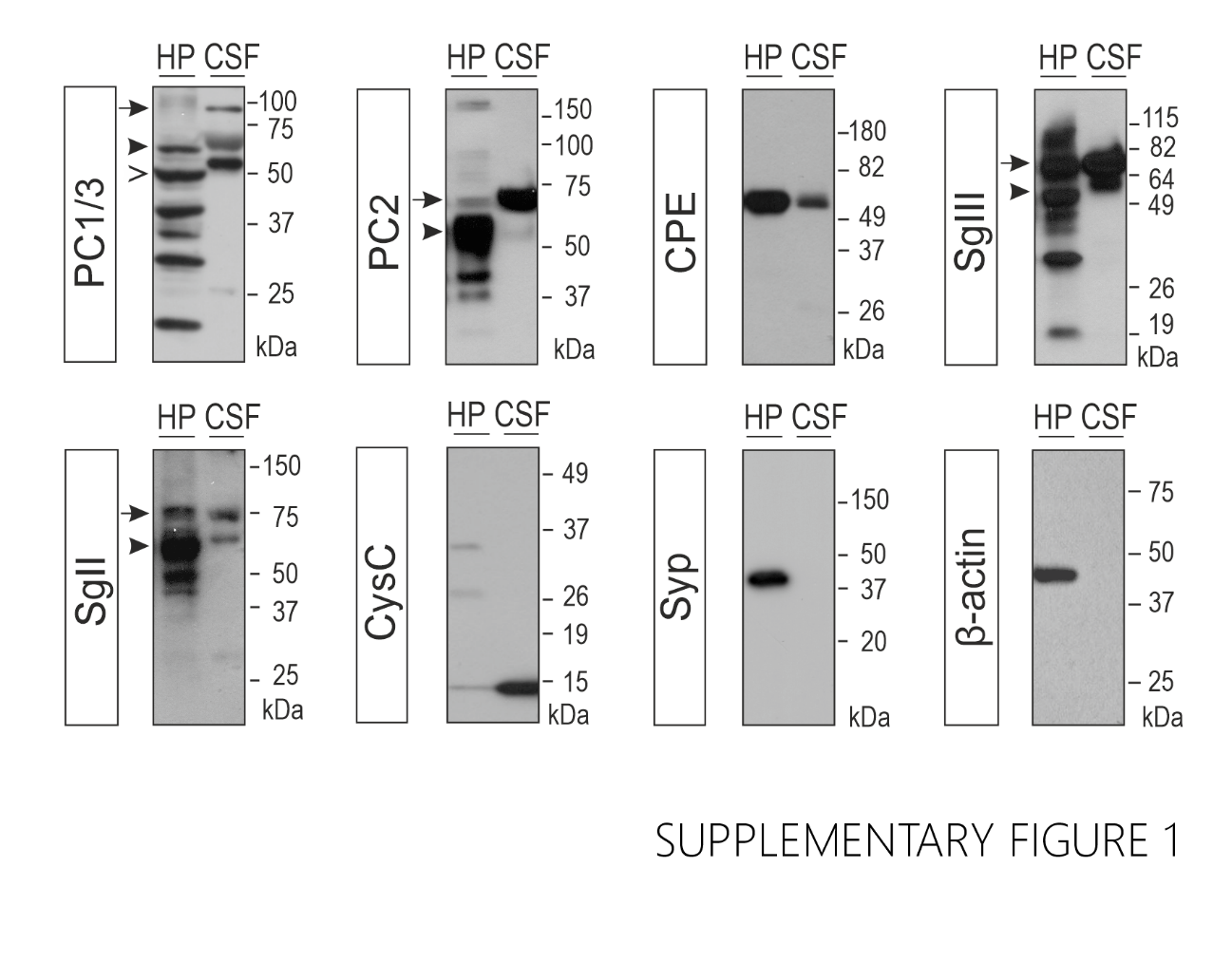
**

**Fig. S1**. **Brain secretory proteins are abundantly detected in human CSF**. Western blotting analysis of the secretory proteins PC1/3, PC2, CPE, SgIII, SgII, and CysC in human hippocampus (hippocampus, 20μg) and CSF (5-10μL). Both samples display the precursor and mature forms (indicated by arrows and arrow-heads, respectively) of granins and convertases, whereas aggregated and cleaved species are essentially detected in brain tissues. CysC monomers (~14 kDa) are present in CSF and hippocampus samples, but only tissues display oligomeric/aggregated forms (~ 28 and ~35 kDa). CSF samples (10μL) lack the membrane and cytosolic proteins synaptophysin (Syp) and β-actin. The mobility of molecular mass markers (in kDa) is indicated.

**
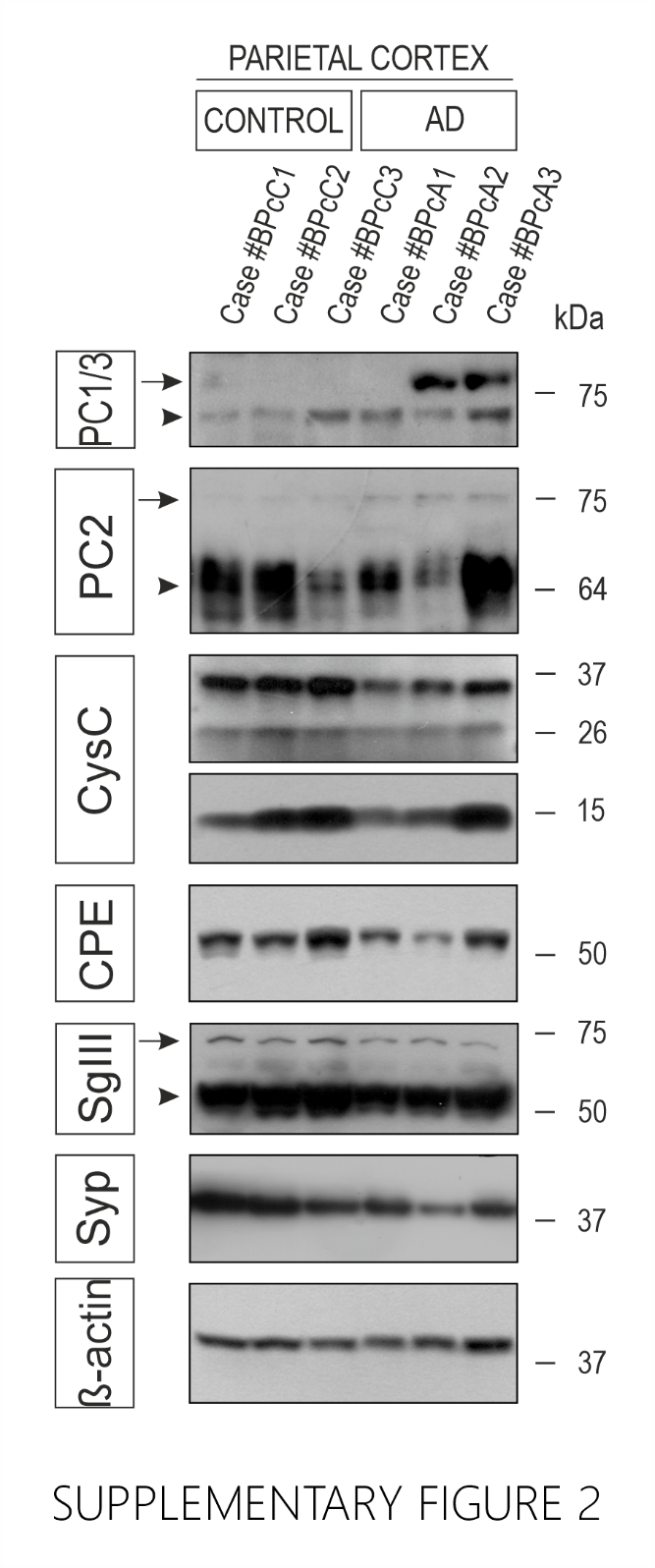
**

**Fig. S2.** **Levels of secretory proteins in the AD parietal cortex.** Representative western blots showing protein levels of PC1/3, PC2, CysC, CPE, SgIII, Syp, and β-actin in homogenates of control and AD paroietal cortex in the same cases. The mobility of molecular mass markers (in kDa) is indicated.


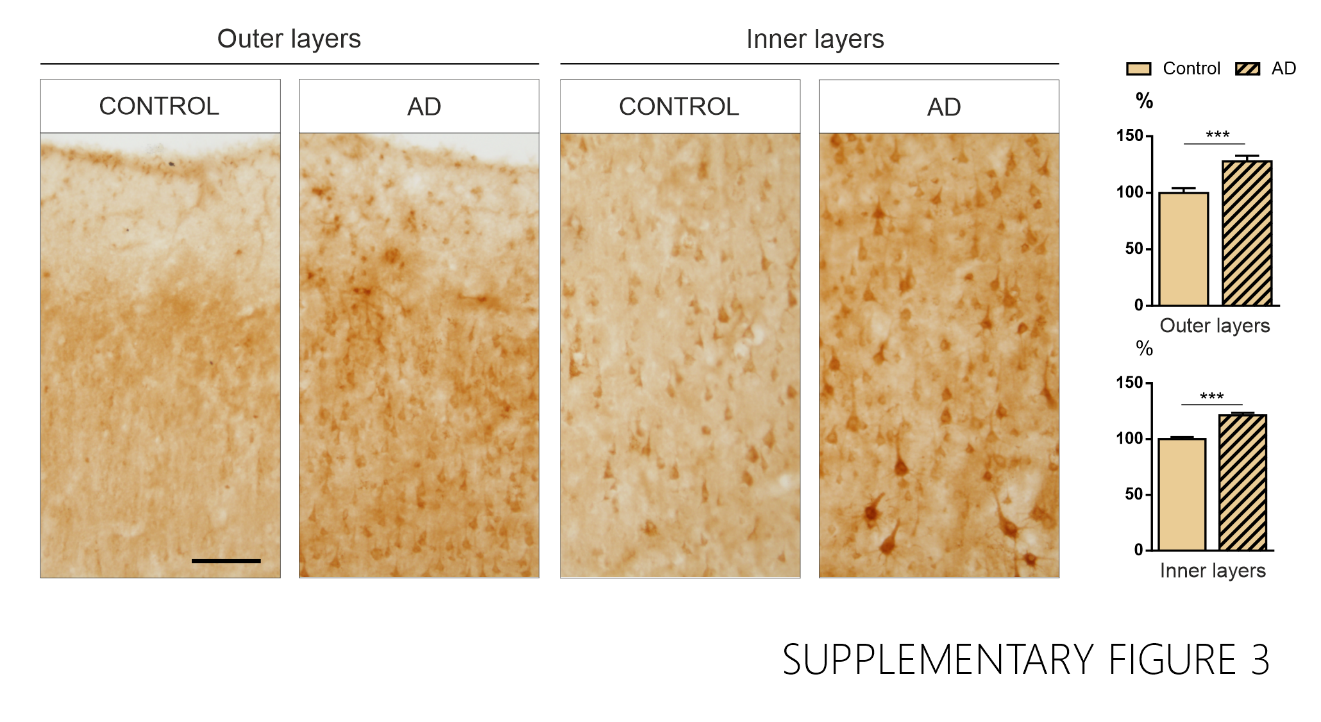


**Fig. S3. Levels and distribution of CysC in the AD parietal cortex.** Left, peroxidase immunohistochemistry for CysC at the outer and inner layers of the AD parietal cortex. Scale bar 50 μm. Right, graphs show the percentage variation compared with controls of the DAB staining intensity. Data are presented as the mean ±SEM. ***p < 0.001, Mann-Whitney test.

| **Table S1. List of used primary antibodies** | | |
| --- | --- | --- |
| Antibody | Source | Reference |
| β actin-peroxidase | Sigma-Aldrich (Diesenhofen, Germany) | Ac-15 |
| Beta-Amyloid | DAKO (Glostrup, Denmark) | 6F/3D |
| CHMP2B | R&D Systems (Minneapolis, MN, USA) | MAB7509 |
| CK1δ | R&D Systems (Minneapolis, MN, USA) | AF4568 |
| CPE | BD Transduction Laboratories (San Jose, CA, USA) | 610758 |
| CPE | GeneTex (Irvine, CA, USA) | GTX11044 |
| CysC | EMD Millipore (Burlington, MA, USA) | ABC20 |
| GFAP | EMD Millipore (Burlington, MA, USA) | MAB360 |
| LAMP1 | Developmental Studies Hybridoma Bank (University of Iowa, Iowa, USA) | H4A3-c |
| NFL | Cell Signalling Technology (Leiden, The Netherlands) | 2835 |
| PC1/3 | Abcam (Cambridge, UK) | ab3532 |
| PC1/3 | Thermo Fisher Scientific (Waltham, MA, USA) | PA1-057 |
| PC2 | Dr. Iris Lindberg (Maryland, BA, USA) | LS18 |
| PC2 | GeneTex (Irvine, CA, USA) | GTX23533 |
| Phospho-Tau | Innogenetics (Gent, Belgium) | AT8 |
| SgII | Dr. Reiner Fischer-Colbrie (Innsbruck, Austria) | SN |
| SgIII | Sigma-Aldrich (Diesenhofen, Germany) | HPA006880 |
| Syp | DAKO Agilent Technologies (Santa Clara, CA, USA) | M 0776 |
